# K^+^ promotes the favorable effect of polyamine on gene expression better than Na^+^

**DOI:** 10.1371/journal.pone.0238447

**Published:** 2020-09-03

**Authors:** Takashi Nishio, Kaito Sugino, Yuko Yoshikawa, Michiaki Matsumoto, Yohei Oe, Koichiro Sadakane, Kenichi Yoshikawa

**Affiliations:** 1 Faculty of Life and Medical Sciences, Doshisha University, Kyoto, Japan; 2 Faculty of Science and Engineering, Doshisha University, Kyoto, Japan; 3 Center for Integrative Medicine and Physics, Institute for Advanced Study, Kyoto University, Kyoto, Japan; Hosei University, JAPAN

## Abstract

**Background:**

Polyamines are involved in a wide variety of biological processes including a marked effect on the structure and function of DNA. During our study on the interaction of polyamines with DNA, we found that K^+^ enhanced *in vitro* gene expression in the presence of polyamine more strongly than Na^+^. Thus, we sought to clarify the physico-chemical mechanism underlying this marked difference between the effects of K^+^ and Na^+^.

**Principal findings:**

It was found that K^+^ enhanced gene expression in the presence of spermidine, SPD(3+), much more strongly than Na^+^, through *in vitro* experiments with a Luciferase assay on cell extracts. Single-DNA observation by fluorescence microscopy showed that Na^+^ prevents the folding transition of DNA into a compact state more strongly than K^+^. ^1^H NMR measurement revealed that Na^+^ inhibits the binding of SPD to DNA more strongly than K^+^. Thus, SPD binds to DNA more favorably in K^+^-rich medium than in Na^+^-rich medium, which leads to favorable conditions for RNA polymerase to access DNA by decreasing the negative charge.

**Conclusion and significance:**

We found that Na^+^ and K^+^ exhibit markedly different effects through competitive binding with a cationic polyamine, SPD, to DNA, which causes a large difference in the higher-order structure of genomic DNA. It is concluded that the larger favorable effect of Na^+^ than K^+^ on *in vitro* gene expression observed in this study is well attributable to the significant difference between Na^+^ and K^+^ on the competitive binding inducing conformational transition of DNA.

## Introduction

Why do living organisms on Earth generally prefer potassium-rich intracellular fluids? Sodium is much more abundant than potassium in sea water [1]. On the contrary, the significantly greater content of potassium ion in the medium of living cells, compared to sodium ion, is a longstanding puzzle. Potassium ion is the major intracellular cation, and exhibits a high specificity over other monovalent cations such as Na^+^. The ionic imbalance of Na^+^ and K^+^ inside and outside the cell is the major factor to determine the membrane potential [2]. It has been argued that K^+^ plays a key role in homeostatic mechanisms such as maintenance of intercellular pH and osmolarity [3–5]. Over the past couple of decades, the generation of a quadruplex structure with guanine-rich sequences, G-quadruplex (G4), has attracted attention from both biological and physicochemical perspectives. G4 is predominantly found in functional regions of the human genome and transcriptome, including replication initiation sites, human telomeres, oncogene promoter regions and untranslated regions [6]. It has been confirmed that K^+^ stabilizes G4 structures much more strongly than Na^+^ [7–13]. K^+^ has also been shown to affect the structure and function of ribosomes [14]. A similar effect of K^+^ was reported in an experiment involving RNA polymerase; i.e., with *E*. *Coli* polymerase the initial reaction velocity is about doubled in the presence of 0.2 M KCl and the synthesis of RNA proceeds for several hours [15]. On the other hand, there is no significant difference between the effects of K^+^ and Na^+^ on the double helix DNA [16–19]. Korolev et al. reported a theoretical model to interpret how monovalent alkali cations cause different effect on the stability of nucleosome, the assembly between DNA and positively-charged histone octamer, by taking into account the effect that Na^+^ has a little bit larger affinity than K^+^ [20].

Recently, we have reported that polyamines can either enhance or inhibit gene expression depending on their concentration [21, 22]. In our study on the effects of polyamine on gene expression, we observed that, in the presence of polyamine, K^+^ enhances gene expression *in vitro* much more strongly than Na^+^. To shed light on this difference in the effects of K^+^ and Na^+^, we measured the effects of K^+^ and Na^+^ on the binding of polyamine to DNA. As the results, it becomes clear that Na^+^ exhibit apparently larger inhibitory effect of polyamine binding to DNA than K^+^. Such observation will be discussed in terms of the greater interfering effect of Na^+^ vs. K^+^ on the binding of polyamine to the phosphate groups of DNA. We will argue that the enhanced gene expression with K^+^ is attributable to its smaller inhibitory effect on polyamine binding. In addition, we discuss the competitive effects of K^+^/Na^+^ on polyamine binding to DNA together with the promotion of gene expression with K^+^, in relation to the intrinsic effect of a K^+^-rich environment in the cell cytoplasm as the general situation in living organisms.

## Materials and methods

### Materials

Spermidine trihydrochloride (SPD) was purchased from Nacalai Tesque (Kyoto Japan). Sodium chloride (NaCl), potassium chloride (KCl) and the antioxidant 2-mercaptoethanol (2-ME) were purchased from FUJIFILM Wako Pure Chemical Corporation (Osaka, Japan). Plasmid DNA (Luciferase T7 Control DNA: 4331 bp) containing both the gene encoding luciferase and the promoter region of T7 RNA polymerase was purchased from Promega (Madison, WI, USA). T4 GT7 bacteriophage DNA (166 kbp with a contour length of 57 μm) was purchased from Nippon Gene (Tokyo, Japan). The dimeric cyanine fluorescent dye YOYO-1 (1,10-(4,4,8,8-tetramethyl-4,8-diazaundecamethylene)bis[4-[(3-methylbenzo-1,3-oxazol-2-yl)methylidene]-l,4-dihydroquinolinium] tetraiodide) was obtained from Molecular Probes Inc. (Eugene, OR, USA). Calf thymus DNA (CT DNA: 8–15 kbp) was purchased from Sigma-Aldrich (St. Louis, MO, USA). Other chemical reagents from commercial sources were of analytical grade.

### Methods

#### Luciferase assay for gene expression

A cell-free *in vitro* luciferase assay was performed with a TnT T7 Quick Coupled Transcription/Translation System (Promega) according to the manufacturer's instructions and previous reports [21, 22]. Plasmid DNA (4331 kbp) was used as the DNA template. The DNA concentration was 0.3 μM in nucleotide units. The reaction mixture containing the DNA template was incubated for 90 min at 30°C on a Dry Thermo Unit (TAITEC, Saitama, Japan). We measured the effects of the addition of various concentrations of spermidine, along with Na^+^ or K^+^ ions, on the luminescence intensity. The expression of luciferase was evaluated following the addition of luciferin as the luciferase substrate (Luciferase Assay Reagent, Promega) by detecting the emission intensity at around 565 nm using a luminometer (MICROTEC Co., Chiba, Japan).

#### Fluorescence Microscopy (FM) observation

To visualize individual DNA molecules in solution by FM, a large DNA, T4 GT7 DNA (166 kbp), was used as described previously [22]. DNA was dissolved in a 10 mM Tris-HCl buffer solution at pH 7.5 with 4% (v/v) 2-ME. For observation of the DNA conformation in solution, desired concentrations of SPD, NaCl and KCl were added to the sample solutions. Measurements were conducted at a low DNA concentration (0.1 μM in nucleotide units) with the addition of YOYO-1 (0.05 μM). Single-molecule observations were performed with an inverted fluorescence microscope (Axiovert 135, Carl Zeiss, Oberkochen, Germany) equipped with a 100× oil-immersion objective lens. Fluorescent illumination was performed using a mercury lamp (100 W) via a filter set (Zeiss-10, excitation BP 450–490; beam splitter FT 510; emission BP 515–565). Images were recorded onto a DVD at 30 frames per second through a high-sensitivity EBCCD (Electron Bombarded Charge-Coupled Device) camera (Hamamatsu Photonics, Shizuoka, Japan) and analyzed with the image-processing software ImageJ (National Institute of Mental Health, MD, USA). Based on the observation of time-successive images, the probability distribution of the long-axis length of DNA in solution was evaluated, and 50 DNA molecules were measured under each experimental condition.

#### NMR titration experiment

The binding abilities of SPD to CT DNA in the presence of Na^+^ or K^+^ were investigated by ^1^H-NMR titration experiments. Nuclear magnetic resonance (NMR) spectra were recorded with a Bruker Ascend 400 spectrometer (400 MHz). All experiments were carried out in 10 mM Tris-DCl buffer (pD 7.5) and 3-(trimethylsilyl)-2,2',3,3'-tetradeuteropropionic acid (TMSP-d4) was used as an internal reference both for chemical shift and signal intensity. The titration samples (0.1 mM SPD and 1.6mM CT DNA) were prepared in 10 mM Tris-DCl buffer (pD 7.5). NaCl and KCl stock solutions were prepared at a concentration of 1000 mM in D_2_O. The titration sample (0.6 mL) was introduced into the NMR tube, and increasing amounts of the titrant NaCl or KCl solution were added.

#### Atomic absorption spectroscopy

The concentrations of Na^+^ and K^+^ contained in the rabbit reticulocyte lysate-based reaction buffer of luciferase assay were measured with an atomic absorption spectrometer, AA-6800 (SHIMADZU, Kyoto, Japan). The measurements were carried out with flame atomization, and a deuterium lamp was used as background correction. The resonance lines of hollow cathode lamps (589.00 nm for Na^+^ and 766.49 nm for K^+^) were used. Calibration curves of Na^+^ and K^+^ (2, 4, 6, 8, 10 ppm) were prepared by diluting the standard solution (1000 ppm) with 0.1 M HCl. Samples were diluted 200 times with 0.1 M HCl. Based on these measurement results, we obtained values of 18 mM and 33 mM, respectively, as the average Na^+^ and K^+^ concentrations in the reaction buffer.

## Results

**Fig 1:**
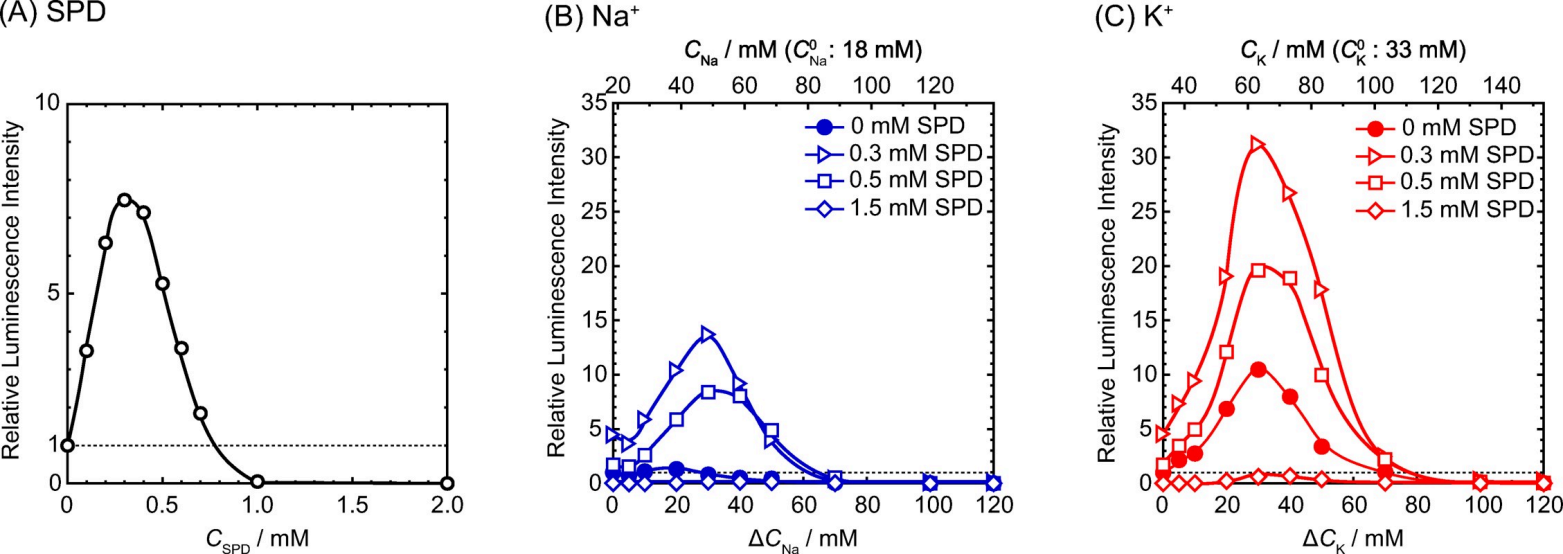
Efficiency of gene expression vs. concentrations of (A) SPD, (B) Na^+^ and (C) K^+^. *C*^0^ is the concentration of Na^+^ and K^+^ contained in the original reaction buffer; *C* = *C*^0^+Δ*C*. The intensity is normalized to the control condition (= 1), where Δ*C* = 0 in the absence of SPD. The DNA concentration was fixed at 0.3 μM.

To study the effects of Na^+^ and K^+^ on gene expression, a cell-free luciferase assay was performed (Fig 1). Fig 1A shows the relative luminescence intensity as a marker of protein synthesis depending on the concentration of SPD. A biphasic effect is observed; i.e., enhancement and inhibition of gene expression at low and higher concentrations, respectively. This biphasic effect of SPD corresponds well to the observations in recent studies [21, 22]. Fig 1B and 1C show the relative luminescence intensity depending on the mole fraction of Na^+^ or K^+^, Δ*C*, added to the reaction buffer at different concentrations of SPD. The upper part of each graph depicts the total concentration *C* of Na^+^ or K^+^, the concentrations *C*^0^ of Na^+^ and K^+^ contained in the original reaction buffer are 18 mM and 33 mM, respectively (see Materials and Methods). The addition of Na^+^ or K^+^ to the solution tends to increase the luminescence intensity, and K^+^ has a greater enhancing effect, except in experiments with 1.5 mM SPD. The complete inhibition at 1.5 mM SPD is attributable to the transition of the higher-order structure to a tightly packed state, as suggested previously [21, 22]. The enhancement of gene expression in the presence of K^+^ has been confirmed at different SPD concentrations as revealed in S1 Fig.

**Fig 2:**
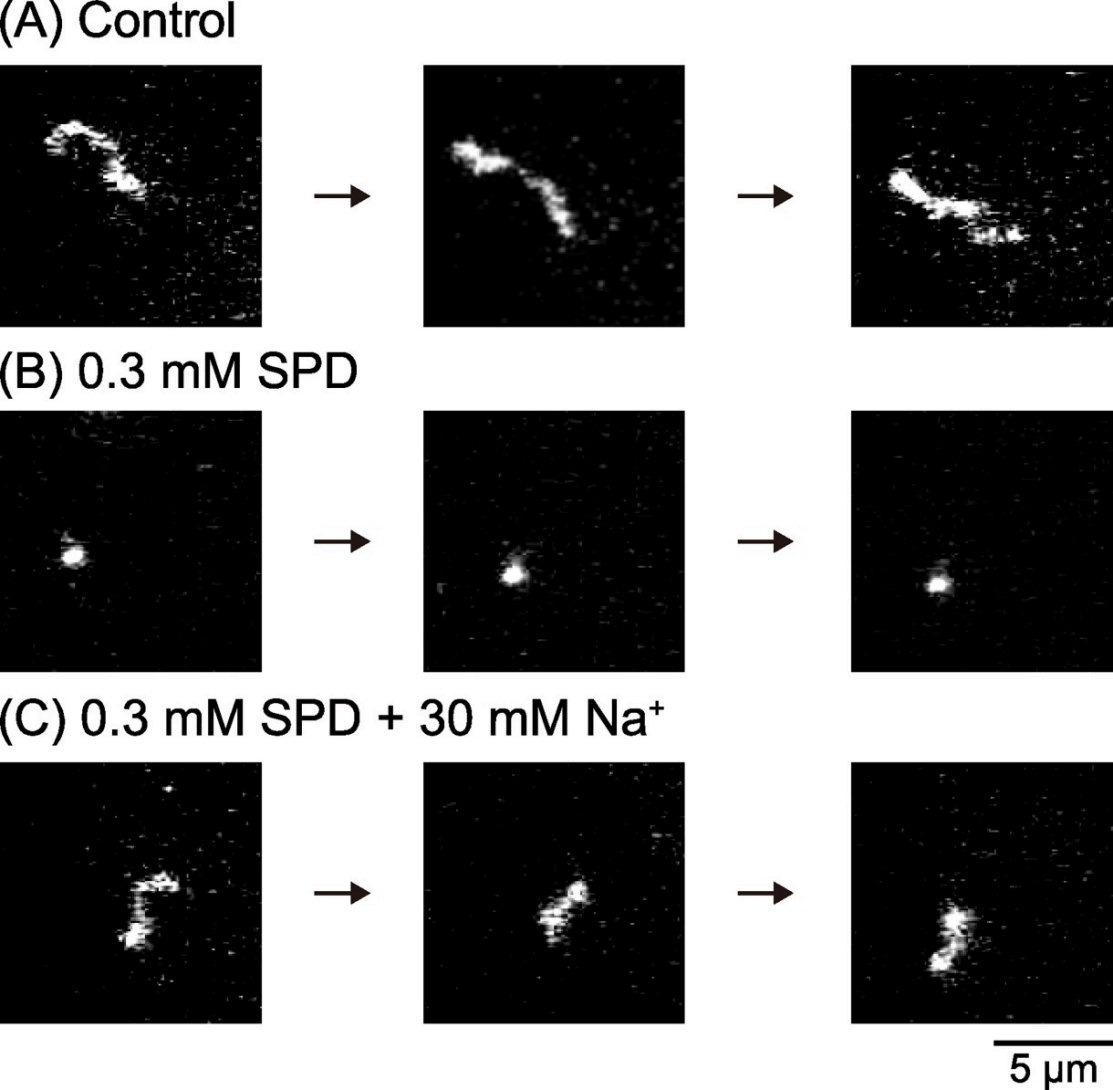
Examples of FM images of a single T4 DNA molecule undergoing Brownian motion in solution. (A) In the absence of any condensation agent such as SPD, Na^+^ or K^+^. (B) In the presence of 0.3 mM SPD. (C) In the presence of 0.3 mM SPD and 30 mM ΔC_Na_. The total observation time for (A)–(C) is 3 s.

To shed light on the effects of Na^+^ and K^+^ on the higher-order structure of DNA in the presence of different concentrations of SPD, we performed real-time single-molecule observation of T4 DNA fluctuating in aqueous solution by using fluorescence microscopy (FM). Fig 2 exemplifies the FM images of single T4 DNA molecules exhibiting translational and intramolecular Brownian motion in aqueous solution. In the absence of SPD, DNA molecules exhibit an elongated random coil conformation (Fig 2A). In the presence of 0.3 mM SPD, a compact globule conformation is observed (Fig 2B). With the addition of 30 mM NaCl to the solution of 0.3 mM SPD, the compact DNA molecule unfolds into an elongated coil conformation (Fig 2C). This retarding effect of K^+^/Na^+^ on the folding transition, or coil-globule transition, corresponds to our past observation [23]. As will be discussed later, the weaker binding of K^+^ to the phosphate group of DNA compared to that of Na^+^ means that K^+^ will have a weaker inhibitory effect to cause the folding transition.

**Fig 3:**
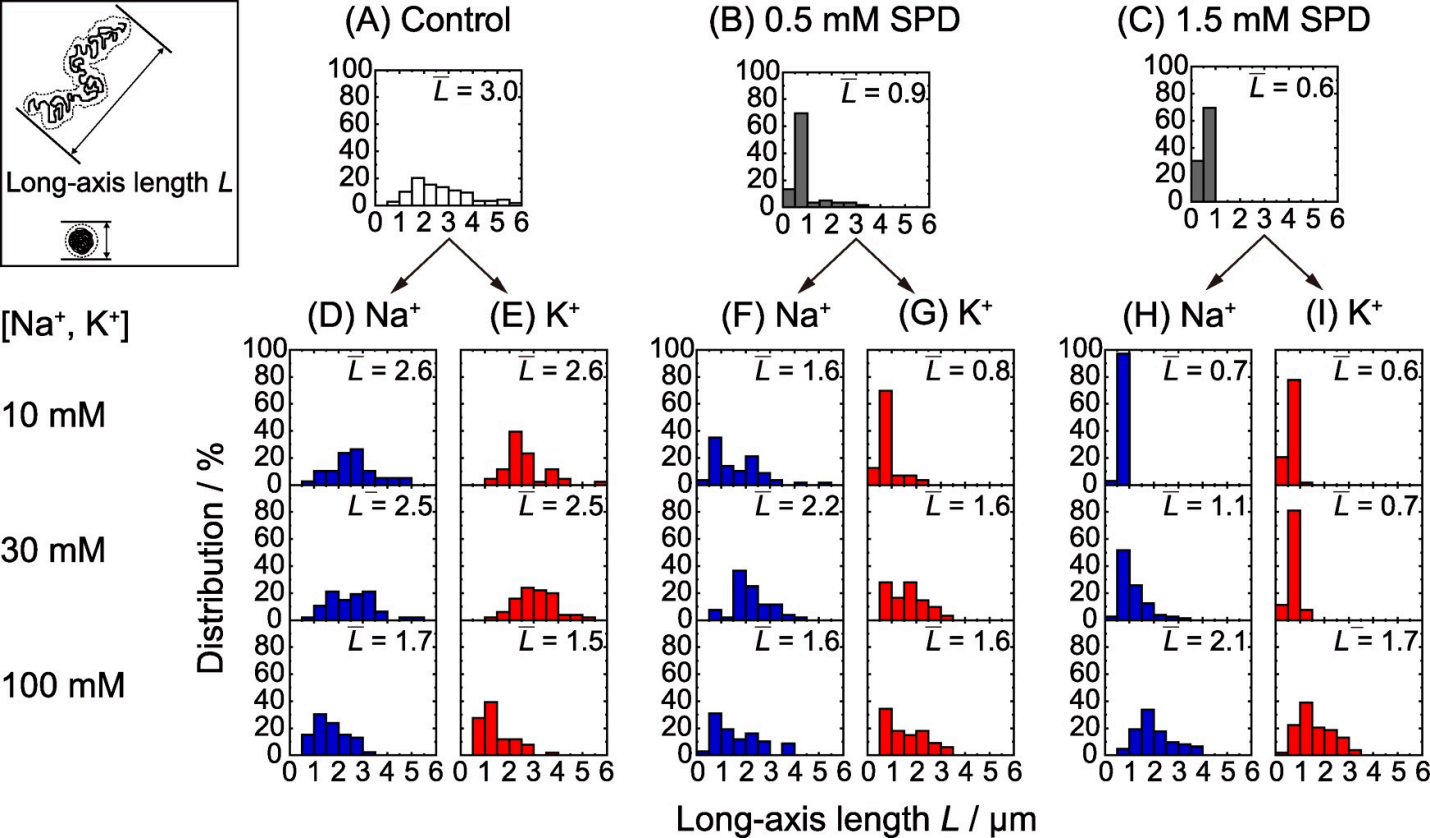
Histograms for the long-axis length *L* of T4 DNA at different concentrations of *C*_Na_ or *C*_K_ with 0–1.5 mM SPD.

Fig 3 shows histograms of the long-axis length *L* of DNA at different concentrations of SPD and at different Na^+^ and K^+^ concentrations, where *L* was evaluated from the FM images of T4 DNA molecules. The long-axis length is represented as a histogram, reflecting the effect of thermal fluctuation of intrachain Brownian motion. Fig 3A–3C reveal the process of the folding transition from an elongated coil to a compact state, i.e., coil-globule transition. In the absence of SPD, Fig 3A shows the elongated state with rather significant fluctuation. With 0.5 mM SPD, the elongated and compact states coexist (Fig 3B). Finally, with 1.5 mM SPD, all of the DNA molecules are in the compact state. Fig 3D–3I show the changes in the long-axis length *L* in the absence of SPD at different concentrations of Na^+^ and K^+^. The histograms in Fig 3D and 3E indicate that DNA molecules tend to shrink slightly, while maintaining the elongated coil state, with an increase in the concentrations of Na^+^ and K^+^. On the other hand, Fig 3F–3I show that both Na^+^ and K^+^ ions tend to unfold compact DNA molecules into the elongated coil state. This inhibitory effect of monovalent cations has been reported in other recent studies and has been interpreted in terms of the competitive effect on the change in the translational entropy of the counter ions [23, 24]. Interestingly, Na^+^ definitely has greater potency for inducing the unfolding transition of DNA in the presence of polyamines.

**Fig 4:**
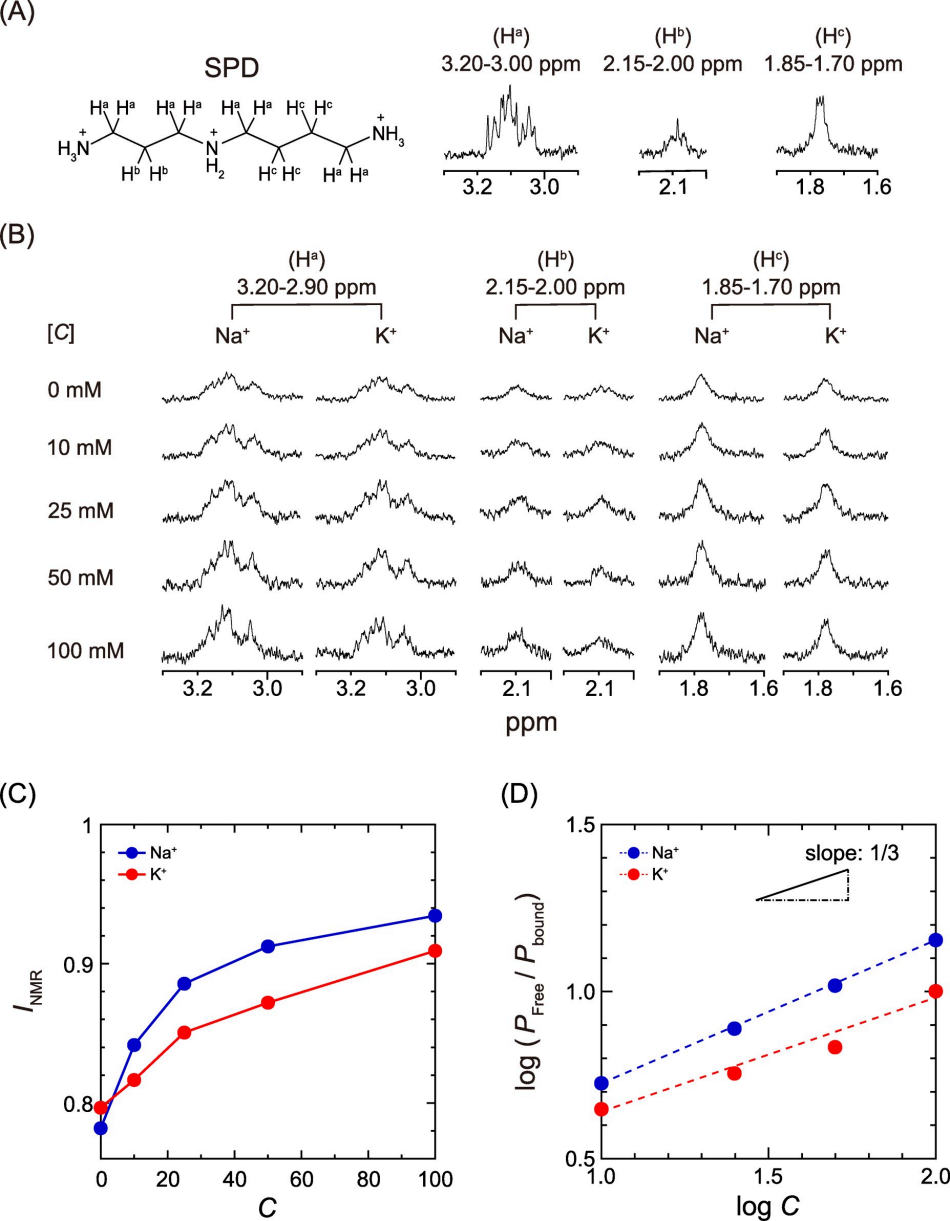
Evaluation of the binding affinity of Na^+^ and K^+^ for DNA through ^1^H NMR measurements. (A) ^1^H NMR signals of 0.1 mM SPD in D_2_O solution. (B) ^1^H NMR signals of 0.1 mM SPD with different concentrations of Na^+^ or K^+^ in the presence of 1.6 mM CT DNA. (C) Changes in the integrated intensity of ^1^H NMR signals, *I*_NMR_, depending on the concentrations *C*. The intensities in the graph were evaluated from the sum of all integrated values for the signals of SPD. (D) Log-log plot; proportion of unbound SPD to bound SPD, *P*_Free/_*P*_Bound_, vs. the salt concentrations *C*. *P*_Free_ and *P*_Bound_ are evaluated from the relationship; *P*_Free_ = *I*_NMR_ and *P*_Bound_ = 1 –*I*_NMR_, respectively.

To evaluate the effect of SPD on the binding affinity of Na^+^ and K^+^ for DNA, we performed ^1^H NMR titration experiments. Fig 4A and 4B show the ^1^H NMR signals of SPD in 10 mM Tris-DCl buffer (pD 7.5) observed at 400 MHz. As shown in Fig 4A, the signals of SPD appear at three different chemical shifts: around δ = 3.20–3.00, 2.15–2.00 and 1.85–1.70 ppm. The assignments of the signals are given in Fig 4A and 4B (for the full ^1^H NMR spectra, see S2 Fig). The top signals in Fig 4B indicate that the intensity of the signals in solutions containing DNA are apparently smaller than those in the absence of DNA as shown in Fig 4A. Such a decrease in signal intensity is attributable to the binding of SPD to DNA, i.e., ^1^H signal of the bound fraction of SPD to DNA is invisible because of a significant decrease of the transverse relaxation time, *T*_2_. [22, 25, 26] With increases in the Na^+^ and K^+^ concentrations, the depressed intensity caused by DNA tends to recover. Such an increase in signal intensity upon the addition of Na^+^ and K^+^ indicates that the degree of dissociation of SPD from DNA increases with an increase in the concentration of monovalent cations [22].

To evaluate the change in the binding affinity of SPD in the presence of Na^+^ and K^+^, Fig 4C shows the intensity of the ^1^H NMR signal of SPD plotted as a function of the Na^+^ or K^+^ concentration. The signal intensities in Fig 4C were calculated based on the sum of all of the integrated values for the spectra of SPD. The graph shows that Na^+^ has a greater effect than K^+^ to suppress the binding degree of SPD to DNA molecules. Fig 4D shows the log-log plot of the data in Fig 4C, revealing almost linear dependences. The slope of the graphs is nearly 1/3, reflecting the effect of the relative magnitudes of the plus-charge between the monovalent cation (Na^+^ and K^+^) and trivalent cation (SPD), as will be discussed in the following section.

## Discussion

In summary, the following points have become clear in the present study: 1) K^+^ enhances gene-expression in the presence of SPD more strongly than Na^+^, based on *in vitro* experiments with a Luciferase assay on cell extracts; 2) Na^+^ suppresses the folding transition of DNA caused by SPD more effectively than K^+^, under a relatively high concentration of SPD with respect to the negatively charged phosphate moiety of DNA; and 3) Na^+^ releases SPD from the binding state to DNA more strongly than K^+^, under a low concentration of SPD with respect to that of the phosphate groups of DNA.

In the present study, we focused on the competitive/cooperative effect of a polyamine, SPD, and monovalent cations, Na^+^ and K^+^, on the DNA structure and genetic activity, since polyamines and monovalent cations naturally coexist in intracellular fluids in living cells. As has been reported previously, Na^+^ tends to bind to the phosphate groups of double-strand DNA somewhat stronger than K^+^ [27, 28]. The results of our study reveal that this difference in binding ability between Na^+^ and K^+^ to the phosphate groups causes an apparent difference in the interaction of polyamine with DNA, as shown in Fig 4. As for the change in the higher-order structure of DNA, it has been revealed that individual DNA molecules undergo a discrete transition from an elongated state to a compact folded state with an increase in polyamines, such as SPD and spermine [21, 23, 29, 30]. The observation of the conformation of single DNA molecules, as reflected in the histogram in Fig 3, indicates the coexistence of elongated and compact states for intermediate concentrations of SPD. Thus, an examination of Fig 3 with respect to the relative ratio between the elongated and compact states indicates that Na^+^ has a greater effect than K^+^ to suppress the formation of the compact state through the decrease of SPD binding to DNA.

It has become evident that Na^+^ inhibits DNA compaction by SPD more strongly than K^+^, based on single-DNA observations in the present study and in a past report [23]. This experimental trend is easily explained in terms of the greater interfering effect of Na^+^ vs. K^+^ on the binding of SPD to the phosphate groups of DNA by inducing the charge neutralization of DNA, as indicated in Fig 4. On the other hand, Na^+^ has higher potential than K^+^ for DNA compaction under a crowded environment in the absence of a polycation such as a polyamine [24]. It is well known that DNA undergoes polymer- and salt-induced (PSI) condensation, Ψ-condensation [31–33]. As reported by Zinchenko et al., for the folding transition of a giant DNA molecule under crowded conditions with polymers from an elongated coil to a compact globule, it has been confirmed that the stronger binding of a monovalent cation as with Na^+^ preferentially causes the compact state, because of the enhancement of the charge neutralization of DNA [24]. In addition, in Fig 4D, the slope is almost 1/3 for the log-log relationship between the monovalent cation concentration and free, or unbound, SPD. This relationship reflects the charge ratio between the monovalent cation and SPD, and is attributable to the competition for the negatively charged phosphate groups of DNA. In relation to the issue of the interaction of polyamine to DNA, Deng, et al. reported that polyamine binds preferentially to DNA phosphate but that binding to major groove of DNA tends to increase with increasing polyamine concentrations [34]. Although such an observation seems to be informative, no experimental data were available for the polyamine concentrations to cause the change of the higher-order structure of DNA, i.e., DNA compaction, because of the experimental difficulty due to the occurrence of precipitation. As will be discussed later, the change of binding position either to phosphate or to electro-negative sites of bases in DNA is expected as an important parameter to describe the different competitive effect of Na^+^ and K^+^ on the interaction of polyamine to DNA.

As noted in the Introduction, the relative binding activities of Na^+^ and K^+^ to DNA are not so different from each other, except in specific cases, such as with four-stranded G4-DNA [7–13]. Such small difference in the manner of binding of monovalent cations to double-stranded DNA has been well explained under the theoretical framework of ‘counterion condensation theory’ [35–38]. This theory suggests that, for double-stranded B-DNA, ca. 76% of the intrinsic negative charges or phosphate groups are neutralized in physiological solutions by attracting monovalent cations from the environment in the absence of multivalent counter cations, and that only 24% remain dissociated to provide a negative charge on DNA [39, 40]. In other words, the degree of counterion condensation around double-strand DNA is dependent mostly on the valency of counter cations and is almost independent of the species of monovalent cations.

Contrary to the expectation deduced from the theory of ‘counter ion condensation’, Na^+^ and K^+^ have dramatically different effects on gene expression as shown in Fig 1. In relation to the effect of monovalent cation on gene expression, Lubin and Ennis reported that, from the experiments by adapting a cell-free system, replace of K^+^ by Na^+^ caused slowing down of protein synthesis [41]. They argued that such an effect was due to depression of transfer rate of amino acid from aminoacyl soluble ribonucleic acid to polypeptide. However, in their study, it was not considered the effect of the structural change in DNA. In the present study, we showed a clear correlation between DNA structural change and gene expression under different Na^+^ and K^+^ concentrations. As such a structural change is mainly caused by polyamines even in the presence of monovalent cations, it is regarded that the interaction of DNA with polyamines plays an essential role in the promotion/inhibition of gene expression (Fig 1B and 1C). Future studies may seek to clarify how polyamines influence RNA and related enzymes, in addition to DNA, during the cell-free luciferase assay to gain a deeper understanding of general kinetic mechanisms in gene expression.

As for the difference between Na^+^ and K^+^ in the interference on the SPD binding to DNA, the mechanism is attributable to the difference in the ionic radius, i.e., the radius of Na^+^ is smaller than that of K^+^ [42]. Studies with NMR measurements have reported that K^+^ exhibits a slightly higher binding affinity to DNA [17, 43]. Additional studies have argued that K^+^ has a slightly higher affinity than Na^+^ for binding to the electronegative sites of DNA bases in the minor and/or major grooves [27, 28]. A study of electrophoretic mobility indicated that Na^+^ exhibits stronger binding to DNA [44]. Other studies have concluded that there is no large difference in the binding constants of Na^+^ and K^+^ to double-strand DNA [17, 27, 45]. On the contrary, we found a significant difference between the effects of Na^+^ and K^+^ on the higher-order structure of DNA in the present and previous studies [23, 24]. The small difference in the nature of binding between Na^+^ and K^+^ may be responsible for the difference in their effects on the higher-order structure of DNA, through competitions of large number of monovalent cations, Na^+^/K^+^, with number of SPD molecules for DNA binding. If we consider DNA of around the size of 100kbp, as in Figs 2 and 3, the number of negative phosphate groups is around 2·10^5^. This indicates that the small difference in the nature of binding of Na^+^ and K^+^ may induce a large difference in the higher-order structural change through the binding of SPD to the larger number of available phosphate groups.

It has been established that giant single DNA molecules above the size of several tens of base-pairs (bp) undergo a large discrete transition between elongated coil and folded compact states, accompanied by a change in density on the order of 10^4^−10^5^ [29, 30]. Thus, we may expect that Na^+^ and K^+^ will have different effects on both the higher-order structure and biological functions of genomic DNA through competition with polycationic species such as polyamines and histones. Allahverdi et al. reported that Na^+^ and K^+^ had opposite effects on nucleosome array folding [46] and on the structural integrity of chromosomes [47]. As for effect of polyamine on *in viro* gene expression, Kanemura et al. reported biphasic effect, acceleration and complete inhibition, depending on the concentration of polyamine [21]. The complete inhibition is caused accompanying by the transition of DNA into a compact state at higher concentration of polyamine. Regarding the acceleration of gene expression at lower concentration of polyamine, a decrease of effective negative charge through the binding of polyamine was suggested to play the major role [21, 22]. The surface of RNA polymerase is highly negatively charged under physiological conditions [48]. Thus, it is expected that polyamines will induce favorable conditions for RNA polymerase to get access to DNA by decreasing negative charge of both DNA and RNA polymerase [21, 22]. There have been several experimental studies on the effect of monovalent cations on the activity of DNA polymerase isolated from different organisms. It was found that the enzymes from both calf thymus and Landschütz ascites tumor cells are stimulated by low concentrations of K^+^ (about 2.5-fold) and Na^+^ (about 1.5-fold), and higher salt concentrations had a considerable inhibitory effect [49]. Goulian et al. found that the activity of the enzyme isolated from *E*. *coli* B infected with bacteriophage T4^+^ was stimulated optimally (1.5-fold) by 50 mM KC1 or NaCl, while higher concentrations of these salts (200 mM) were strongly inhibitory, and there seemed to be no remarkable difference between K^+^ and Na^+^ [50]. However, it was reported that K^+^ stimulates DNA polymerase of *E*. *Coli* B slightly more strongly than Na^+^ [51]. The results regarding the effects of monovalent cations on DNA polymerase imply that the difference between the effects of K^+^ and Na^+^ on gene expression reported here is most probably attributable to the small difference in the binding strength of K^+^/Na^+^ for the large number of phosphates on giant DNA molecules with respect to competition with polyamine binding.

More than a half-century ago, Evans and Sorger stated the generalization that a large number of enzymes are activated by K^+^ [52]. Since then many studies have focused on how K^+^ and Na+ have different effects on the structure and function of individual enzymes [53, 54] and negatively-charged polypeptides [55, 56]. Here, we clarified that the competitive interaction with polyamines underlies the large difference between the effects of K^+^ and Na^+^, although there is very little difference in the nature of the interaction of these monovalent cations with DNA. Living organisms on Earth are adapted to a K^+^-rich intracellular solution. The significant effect of competition of monovalent cations with other biochemical species as reported in the present study may provide a new insight into the strategy of living organisms to use such a K^+^-rich medium, in relation to the longstanding problem of the ‘origin of life’ [5, 57].

In the present study, it has become clear that Na^+^ and K^+^ have markedly different effects on the binding of a cationic polyamine, SPD, to double-strand DNA and, consequently, also on the higher-order structure of giant DNA. This significant difference between Na^+^ and K^+^ is expected to be associated with the general trend that living cells on Earth tend to favor K^+^-rich intracellular medium. The finding that Na^+^ more strongly interferes with the binding of SPD to DNA is attributable to its stronger binding to phosphate groups of DNA because of the smaller ionic radius of Na^+^. On the other hand, it has been considered that there is almost no difference between Na^+^ and K^+^ regarding the degree of counter ion condensation along double-stranded DNA as a strongly charged polyelectrolyte. The large difference in the effects of Na^+^ and K^+^ on *in vitro* gene expression is attributable to the different interference effects of these monovalent cations on the binding of polyamine to DNA, at least as the main cause. Further studies on the biological effects of the competition between monovalent cations and polyamine will shed light on the longstanding unsolved problem concerning the selectivity between Na^+^ and K^+^ in living systems.

## Supporting information

S1 Fig Gene expression efficiency depending on SPD concentration. Closed circle: without addition of Na^+^ and K^+^. Blue open circle: with addition of 30 mM Na^+^ (Δ*C_Na_*) to the reaction buffer. Red open circle: with addition of 30 mM K^+^ (Δ*C_K_*) to the reaction buffer. The concentrations of Na^+^ and K^+^ contained in the original rabbit reticulocyte lysate-based reaction buffer is 18 mM and 33 mM, respectively. DNA concentration was fixed at 0.3 μM.
(PDF)

S2 Fig 1H NMR parameters and spectra. (PDF)
