# Supplementary figures and images for: K+ promotes the favorable effect of polyamine on gene expression better than Na+
Source: PLoS One. 2020 Sep 3;15(9):e0238447. doi: 10.1371/journal.pone.0238447 (PMC7470421; doi:10.1371/journal.pone.0238447)

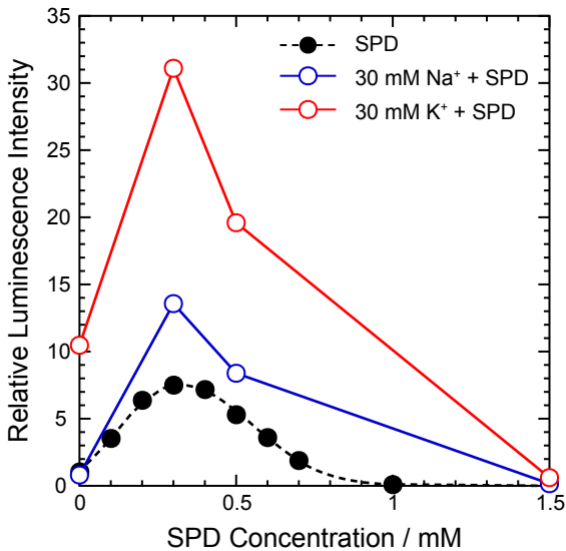

Supplement: S1 Fig — Closed circle: without addition of Na+ and K+. Blue open circle: with addition of 30 mM Na+ (ΔCNa) to the reaction buffer. Red open circle: with addition of 30 mM K+ (ΔCK) to the reaction buffer. The concentrations of Na+ and K+ contained in the original rabbit reticulocyte lysate-based reaction buffer is 18 mM and 33 mM, respectively. DNA concentration was fixed at 0.3 μM. (PDF) [file pone.0238447.s001.pdf]
