# Supplementary material for: K+ promotes the favorable effect of polyamine on gene expression better than Na+
Source: PLoS One. 2020 Sep 3;15(9):e0238447. doi: 10.1371/journal.pone.0238447 (PMC7470421; doi:10.1371/journal.pone.0238447)
Supplement: S2 Fig — (PDF) [file pone.0238447.s002.pdf]

(a) 0.1 mM SPD

^{1}H NMR parameters

The following parameters were adapted.
NMR instrument: Bruker Ascend 400 spectrometer
Observation frequency: 400 MHz
Nucleus: ^{1}H
Acquisition time: 4.09 s
Number of scans: 64
Temperature: 24.8 °C
Solvent: D_2O with 10mM Tris-DCl buffer (pD 7.5)
Internal standard of chemical shift: TMSP-d4 (δ = 0 ppm)

^{1}H NMR Spectrum

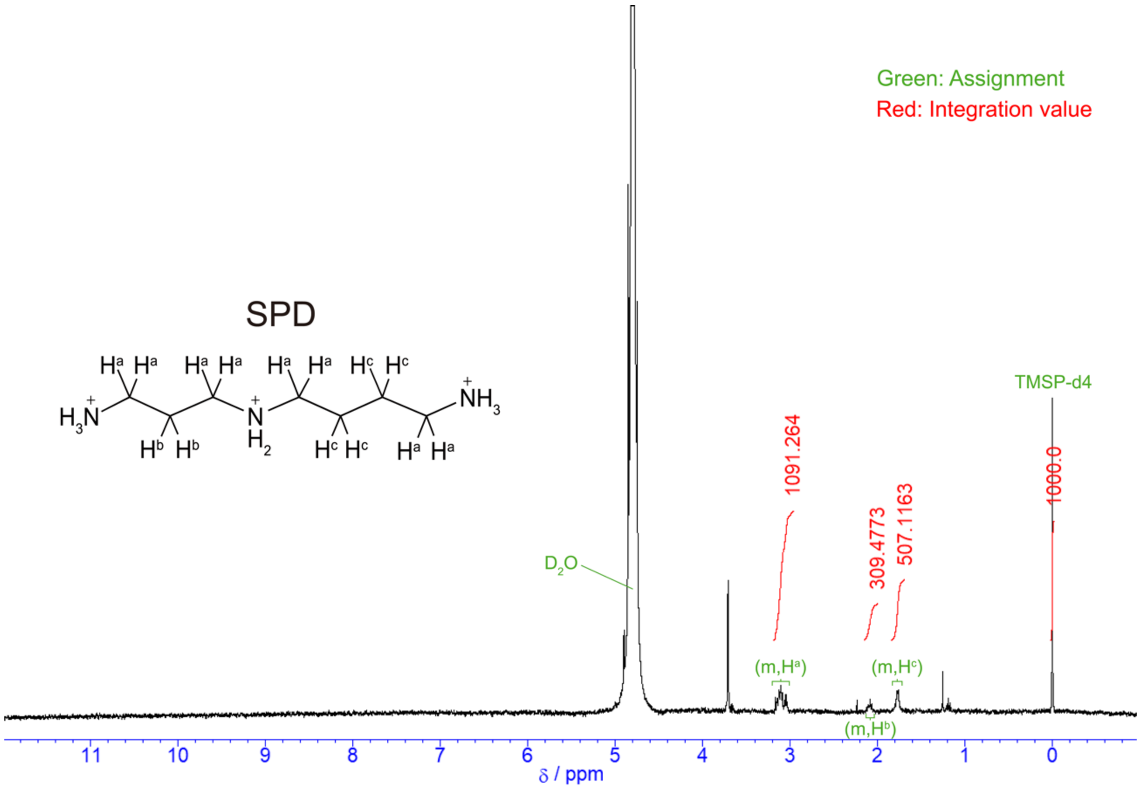

The chemical shift assignments of spermidine were performed manually by extracting spectral information from the HMDB [1].
(https://www.hmdb.ca/spectra/nmr_one_d/1671)

Spermidine: ^{1}H NMR (400 MHz, D_2O): δ 1.70-1.85 (m, 4H), 2.00-2.15 (m, 2H), 3.00-3.20 (m, 8H) ppm. CAS registry No: 124-20-9

(b) 0.1 mM SPD + 1.6 mM CT DNA + 0 mM NaCl

^{1}H NMR parameters

The following parameters were adapted.

NMR instrument: Bruker Ascend 400 spectrometer

Observation frequency: 400 MHz

Nucleus: ^{1}H

Acquisition time: 4.09 s

Number of scans: 64

Temperature: 24.8 °C

Solvent: D_2O with 10mM Tris-DCl buffer (pD 7.5)

Internal standard of chemical shift: TMSP-d4 (δ = 0 ppm)

^{1}H NMR Spectrum

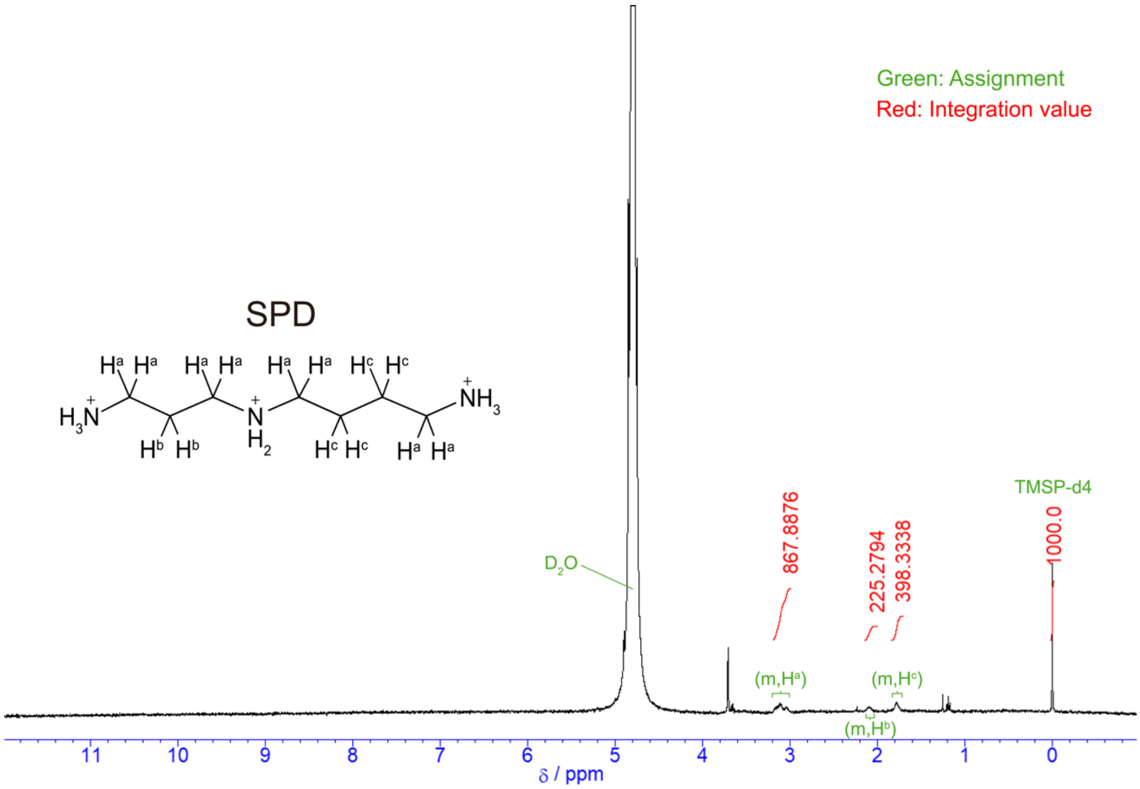

The chemical shift assignments of spermidine were performed manually by extracting spectral information from the HMDB [1]. (https://www.hmdb.ca/spectra/nmr_one_d/1671)

Spermidine: ^{1}H NMR (400 MHz, D_2O): δ 1.70-1.85 (m, 4H), 2.00-2.15 (m, 2H), 3.00-3.20 (m, 8H) ppm. CAS registry No: 124-20-9

(c) 0.1 mM SPD + 1.6 mM CT DNA + 10 mM NaCl

^{1}H NMR parameters

The following parameters were adapted.

NMR instrument: Bruker Ascend 400 spectrometer

Observation frequency: 400 MHz

Nucleus: ^{1}H

Acquisition time: 4.09 s

Number of scans: 64

Temperature: 24.8 °C

Solvent: D_2O with 10mM Tris-DCl buffer (pD 7.5)

Internal standard of chemical shift: TMSP-d4 (δ = 0 ppm)

^{1}H NMR Spectrum

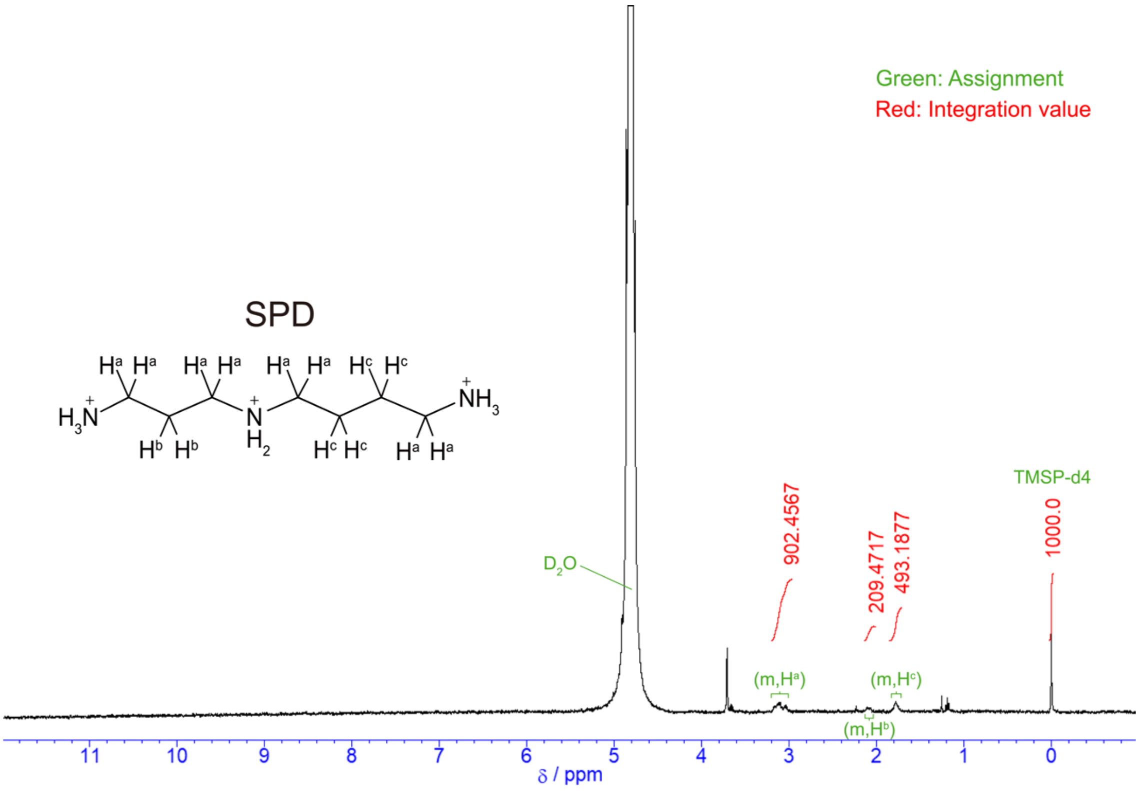

The chemical shift assignments of spermidine were performed manually by extracting spectral information from the HMDB [1]. (https://www.hmdb.ca/spectra/nmr_one_d/1671)

Spermidine: ^{1}H NMR (400 MHz, D_2O): δ 1.70-1.85 (m, 4H), 2.00-2.15 (m, 2H), 3.00-3.20 (m, 8H) ppm. CAS registry No: 124-20-9

(d) 0.1 mM SPD + 1.6 mM CT DNA + 25 mM NaCl

^{1}H NMR parameters

The following parameters were adapted.
NMR instrument: Bruker Ascend 400 spectrometer
Observation frequency: 400 MHz
Nucleus: ^{1}H
Acquisition time: 4.09 s
Number of scans: 64
Temperature: 24.8 °C
Solvent: D_2O with 10mM Tris-DCl buffer (pD 7.5)
Internal standard of chemical shift: TMSP-d4 (δ = 0 ppm)

^{1}H NMR Spectrum

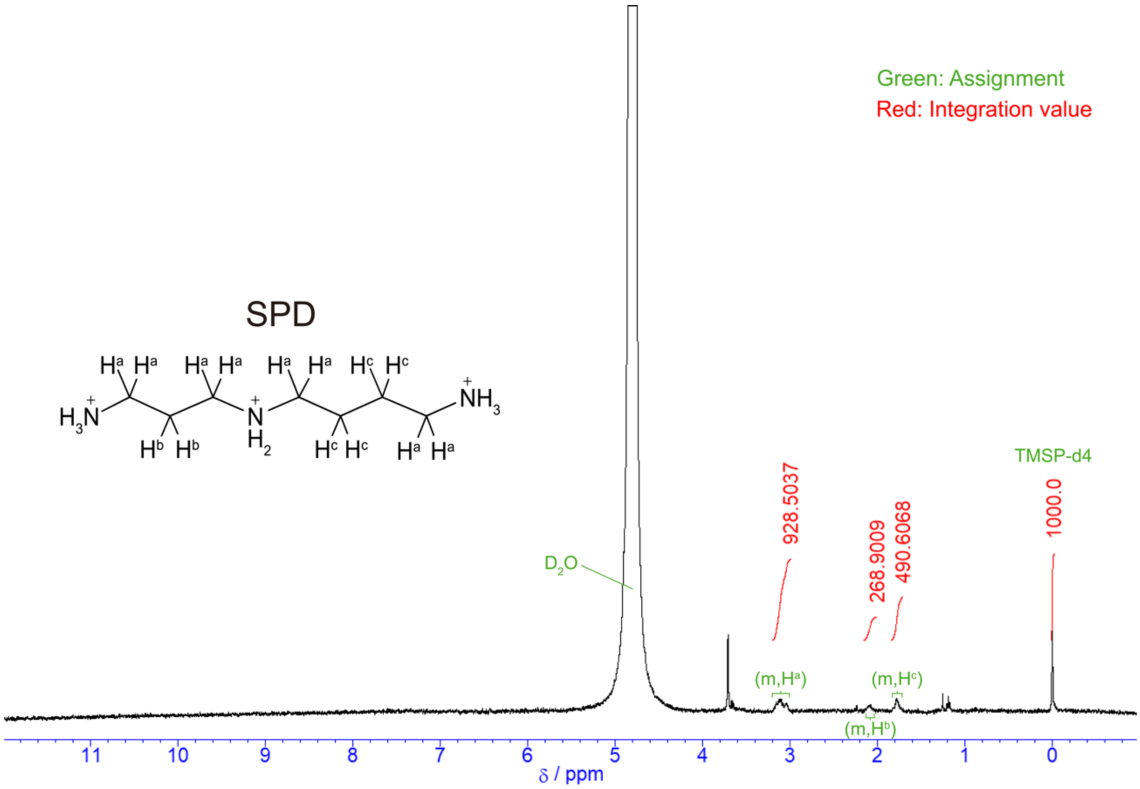

The chemical shift assignments of spermidine were performed manually by extracting spectral information from the HMDB [1].
(https://www.hmdb.ca/spectra/nmr_one_d/1671)

Spermidine: ^{1}H NMR (400 MHz, D_2O): δ 1.70-1.85 (m, 4H), 2.00-2.15 (m, 2H), 3.00-3.20 (m, 8H) ppm. CAS registry No: 124-20-9

(e) 0.1 mM SPD + 1.6 mM CT DNA + 50 mM NaCl

^{1}H NMR parameters

The following parameters were adapted.

NMR instrument: Bruker Ascend 400 spectrometer

Observation frequency: 400 MHz

Nucleus: ^{1}H

Acquisition time: 4.09 s

Number of scans: 64

Temperature: 24.8 °C

Solvent: D_2O with 10mM Tris-DCl buffer (pD 7.5)

Internal standard of chemical shift: TMSP-d4 (δ = 0 ppm)

^{1}H NMR Spectrum

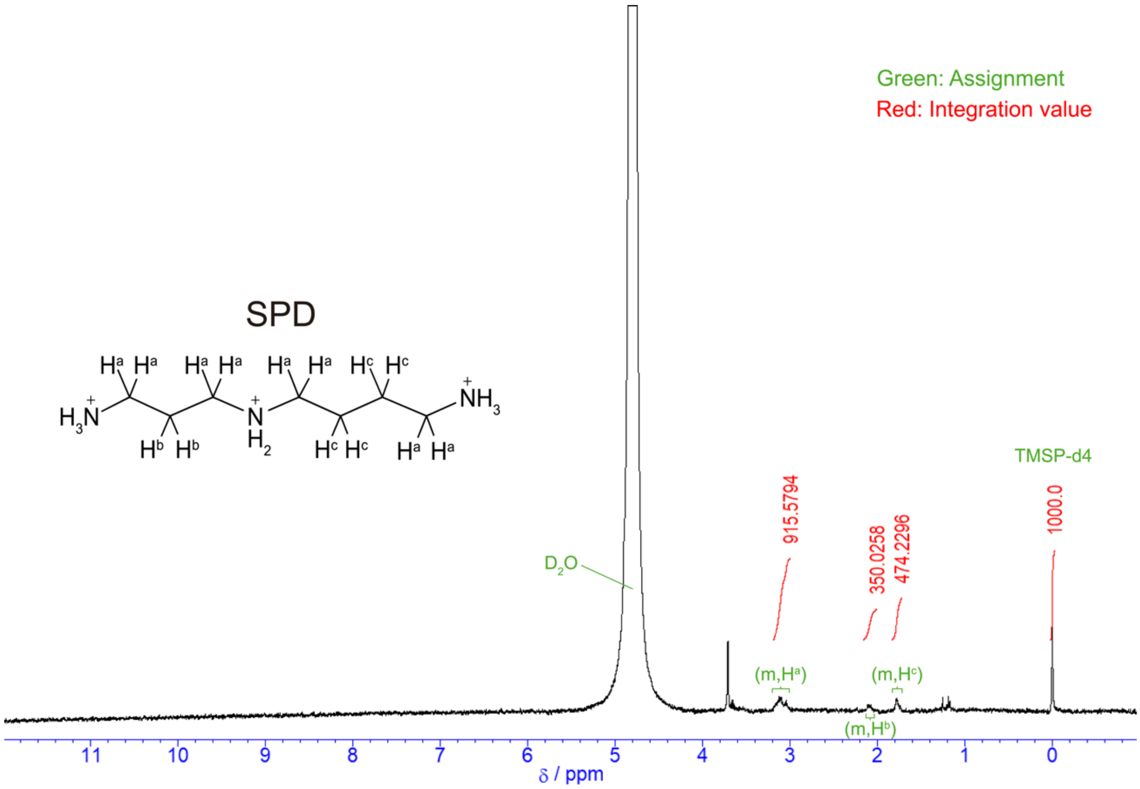

The chemical shift assignments of spermidine were performed manually by extracting spectral information from the HMDB [1]. (https://www.hmdb.ca/spectra/nmr_one_d/1671)

Spermidine: ^{1}H NMR (400 MHz, D_2O): δ 1.70-1.85 (m, 4H), 2.00-2.15 (m, 2H), 3.00-3.20 (m, 8H) ppm. CAS registry No: 124-20-9

(f) 0.1 mM SPD + 1.6 mM CT DNA + 100 mM NaCl

^{1}H NMR parameters

The following parameters were adapted.

NMR instrument: Bruker Ascend 400 spectrometer

Observation frequency: 400 MHz

Nucleus: ^{1}H

Acquisition time: 4.09 s

Number of scans: 64

Temperature: 24.8 °C

Solvent: D_2O with 10mM Tris-DCl buffer (pD 7.5)

Internal standard of chemical shift: TMSP-d4 (δ = 0 ppm)

^{1}H NMR Spectrum

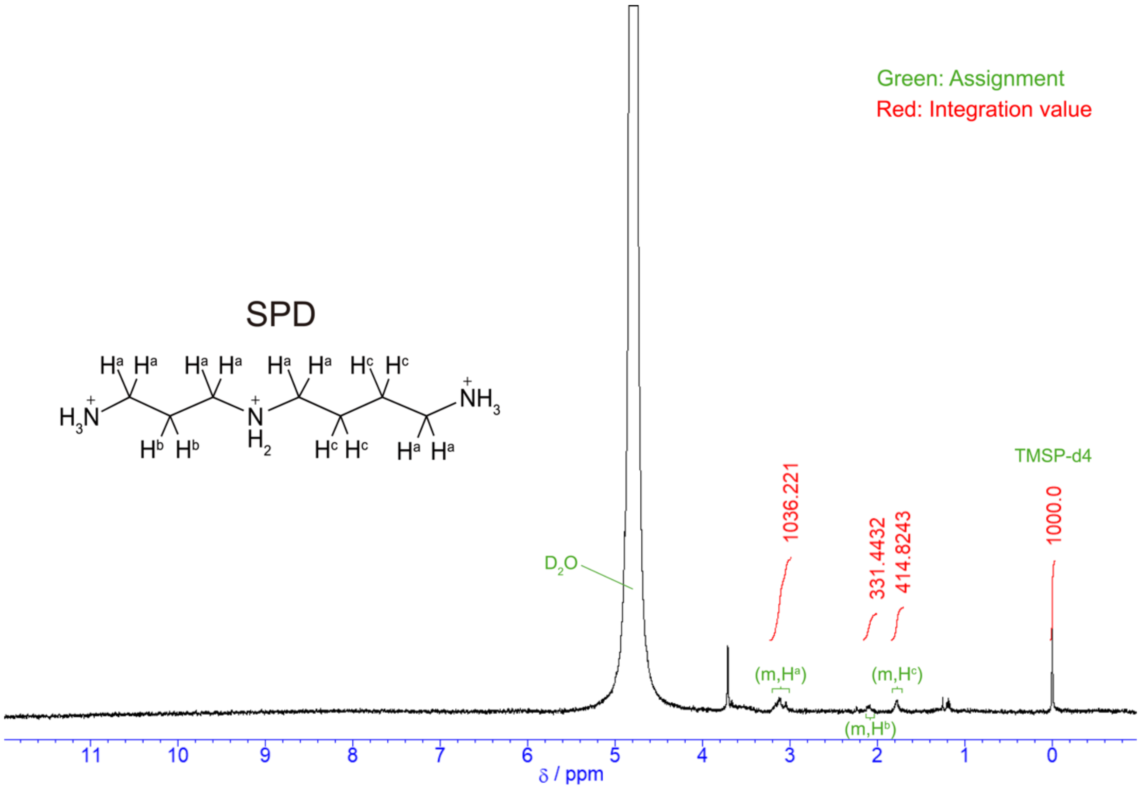

The chemical shift assignments of spermidine were performed manually by extracting spectral information from the HMDB [1]. (https://www.hmdb.ca/spectra/nmr_one_d/1671)

Spermidine: ^{1}H NMR (400 MHz, D_2O): δ 1.70-1.85 (m, 4H), 2.00-2.15 (m, 2H), 3.00-3.20 (m, 8H) ppm. CAS registry No: 124-20-9

(g) 0.1 mM SPD + 1.6 mM CT DNA + 0 mM KCl

^{1}H NMR parameters

The following parameters were adapted.

NMR instrument: Bruker Ascend 400 spectrometer

Observation frequency: 400 MHz

Nucleus: ^{1}H

Acquisition time: 4.09 s

Number of scans: 64

Temperature: 24.8 °C

Solvent: D_2O with 10mM Tris-DCl buffer (pD 7.5)

Internal standard of chemical shift: TMSP-d4 (δ = 0 ppm)

^{1}H NMR Spectrum

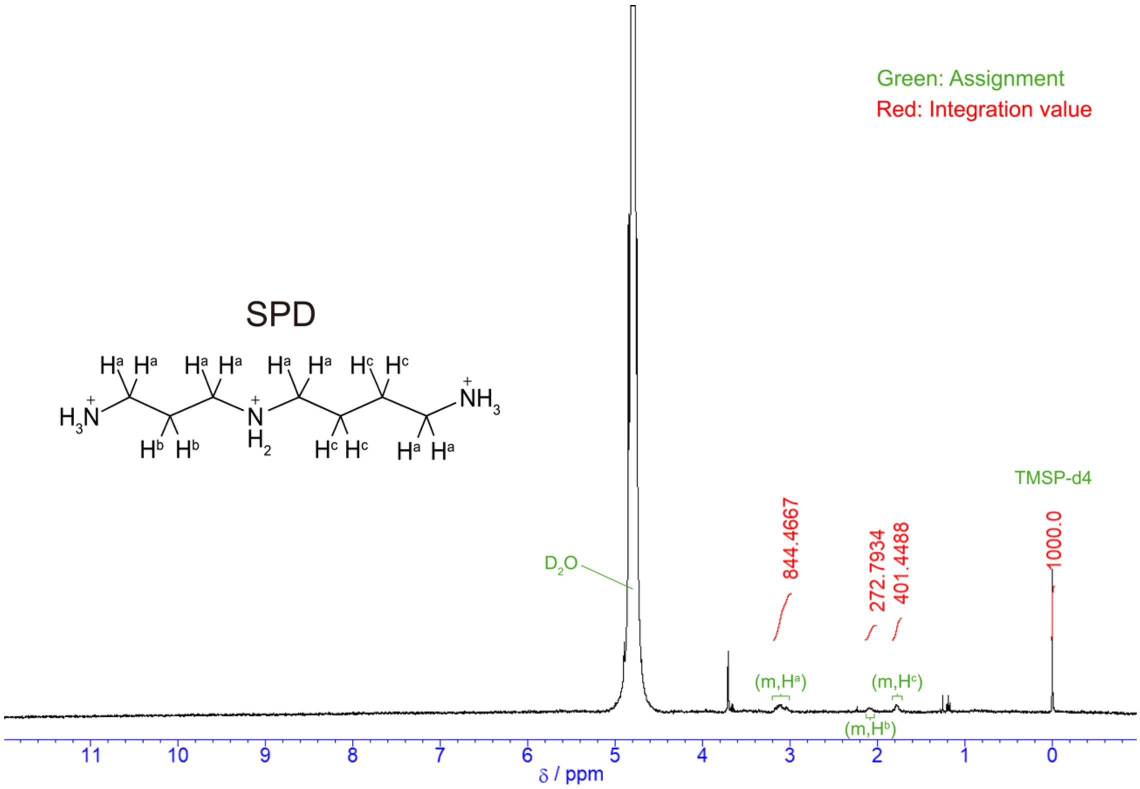

The chemical shift assignments of spermidine were performed manually by extracting spectral information from the HMDB [1]. (https://www.hmdb.ca/spectra/nmr_one_d/1671)

Spermidine: ^{1}H NMR (400 MHz, D_2O): δ 1.70-1.85 (m, 4H), 2.00-2.15 (m, 2H), 3.00-3.20 (m, 8H) ppm. CAS registry No: 124-20-9

(h) 0.1 mM SPD + 1.6 mM CT DNA + 10 mM NaCl

^{1}H NMR parameters

The following parameters were adapted.

NMR instrument: Bruker Ascend 400 spectrometer

Observation frequency: 400 MHz

Nucleus: ^{1}H

Acquisition time: 4.09 s

Number of scans: 64

Temperature: 24.8 °C

Solvent: D_2O with 10mM Tris-DCl buffer (pD 7.5)

Internal standard of chemical shift: TMSP-d4 (δ = 0 ppm)

^{1}H NMR Spectrum

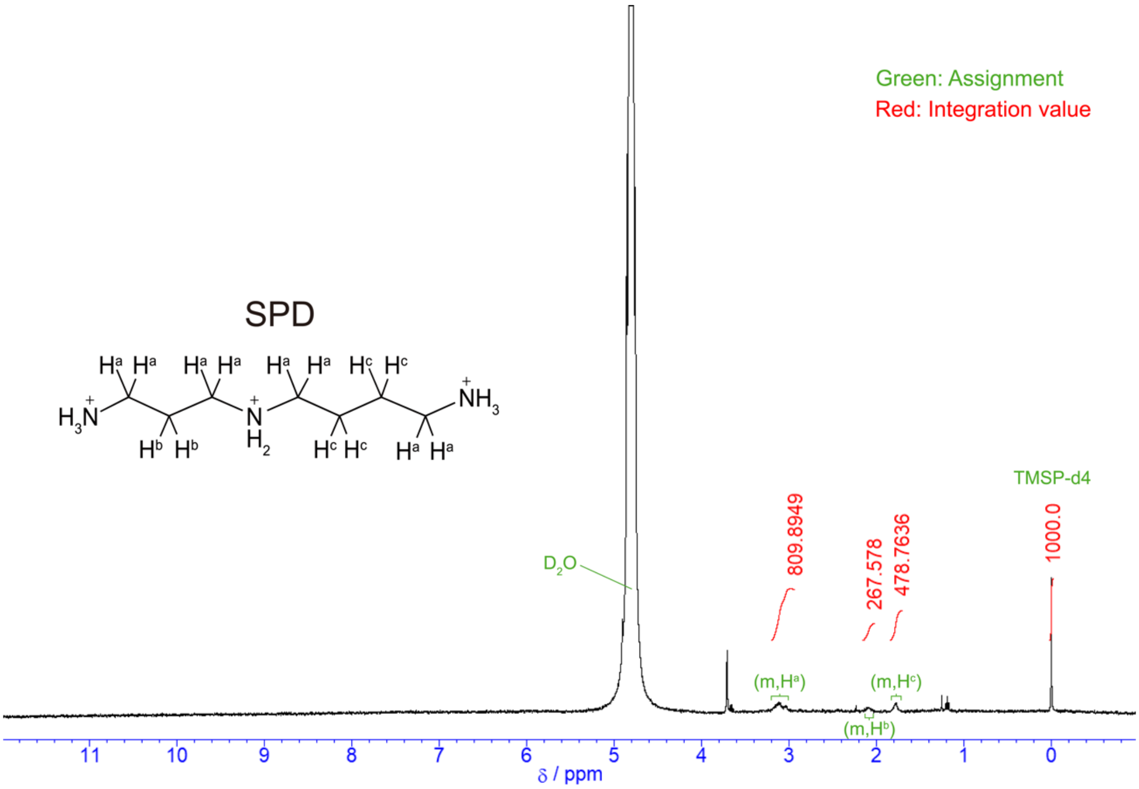

The chemical shift assignments of spermidine were performed manually by extracting spectral information from the HMDB [1].
(https://www.hmdb.ca/spectra/nmr_one_d/1671)

Spermidine: ^{1}H NMR (400 MHz, D_2O): δ 1.70-1.85 (m, 4H), 2.00-2.15 (m, 2H), 3.00-3.20 (m, 8H) ppm. CAS registry No: 124-20-9

(i) 0.1 mM SPD + 1.6 mM CT DNA + 25 mM NaCl

^{1}H NMR parameters

The following parameters were adapted.

NMR instrument: Bruker Ascend 400 spectrometer

Observation frequency: 400 MHz

Nucleus: ^{1}H

Acquisition time: 4.09 s

Number of scans: 64

Temperature: 24.8 °C

Solvent: D_2O with 10mM Tris-DCl buffer (pD 7.5)

Internal standard of chemical shift: TMSP-d4 ($\delta = 0$ ppm)

^{1}H NMR Spectrum

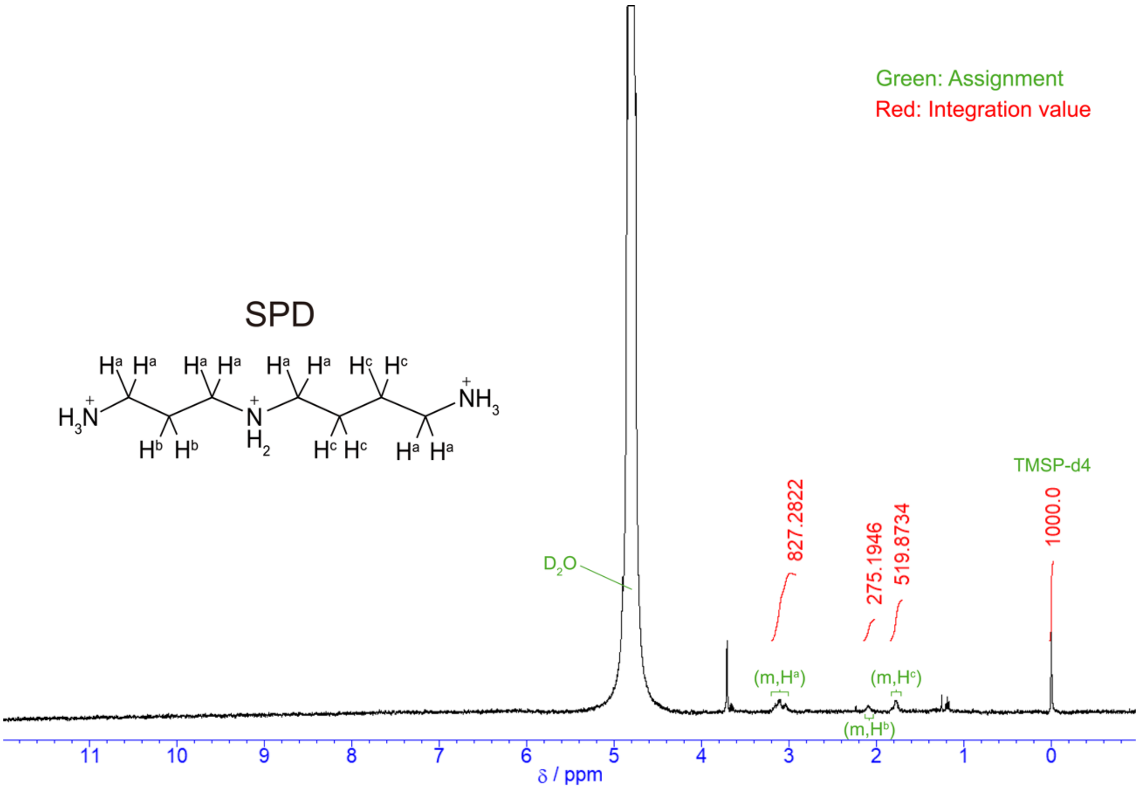

The chemical shift assignments of spermidine were performed manually by extracting spectral information from the HMDB [1]. (https://www.hmdb.ca/spectra/nmr_one_d/1671)

Spermidine: ^{1}H NMR (400 MHz, D_2O): δ 1.70-1.85 (m, 4H), 2.00-2.15 (m, 2H), 3.00-3.20 (m, 8H) ppm. CAS registry No: 124-20-9

(j) 0.1 mM SPD + 1.6 mM CT DNA + 50 mM NaCl

^{1}H NMR parameters

The following parameters were adapted.

NMR instrument: Bruker Ascend 400 spectrometer

Observation frequency: 400 MHz

Nucleus: ^{1}H

Acquisition time: 4.09 s

Number of scans: 64

Temperature: 24.8 °C

Solvent: D_2O with 10mM Tris-DCl buffer (pD 7.5)

Internal standard of chemical shift: TMSP-d4 (δ = 0 ppm)

^{1}H NMR Spectrum

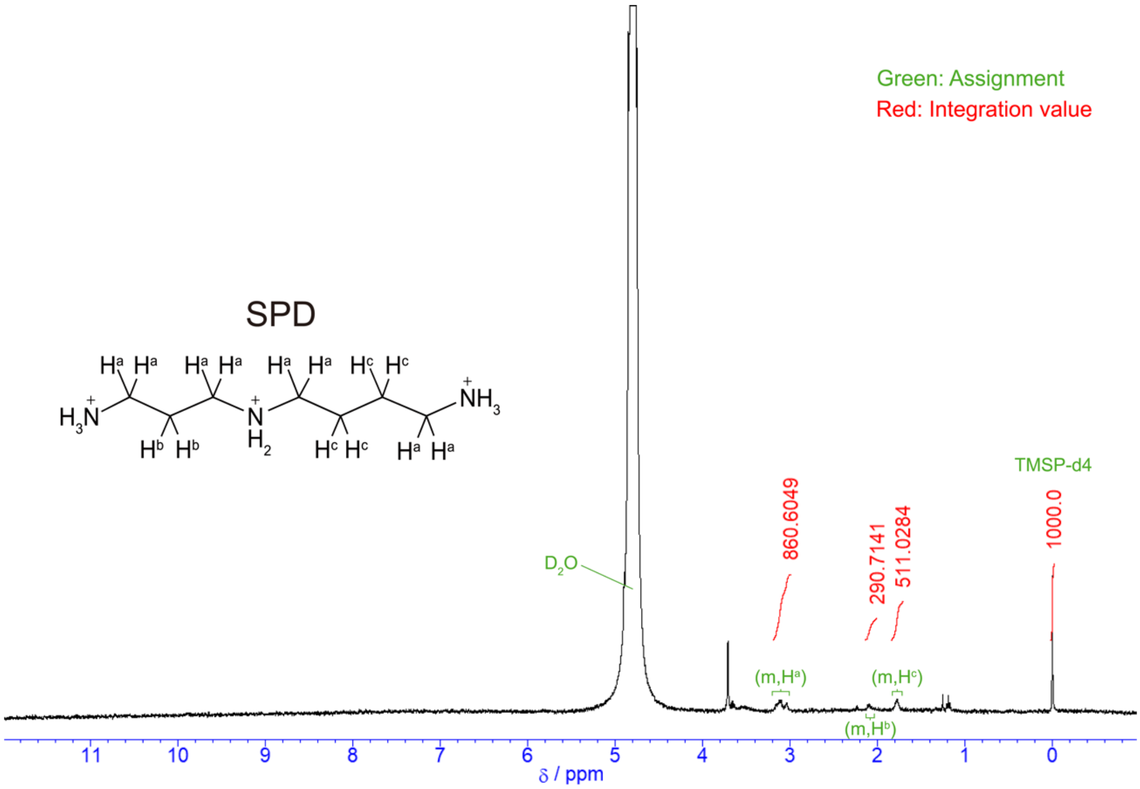

The chemical shift assignments of spermidine were performed manually by extracting spectral information from the HMDB [1].

(https://www.hmdb.ca/spectra/nmr_one_d/1671)

Spermidine: ^{1}H NMR (400 MHz, D_2O): δ 1.70-1.85 (m, 4H), 2.00-2.15 (m, 2H), 3.00-3.20 (m, 8H) ppm. CAS registry No: 124-20-9

(k) 0.1 mM SPD + 1.6 mM CT DNA + 100 mM NaCl

^{1}H NMR parameters

The following parameters were adapted.

NMR instrument: Bruker Ascend 400 spectrometer

Observation frequency: 400 MHz

Nucleus: ^{1}H

Acquisition time: 4.09 s

Number of scans: 64

Temperature: 24.8 °C

Solvent: D_2O with 10mM Tris-DCl buffer (pD 7.5)

Internal standard of chemical shift: TMSP-d4 (δ = 0 ppm)

^{1}H NMR Spectrum

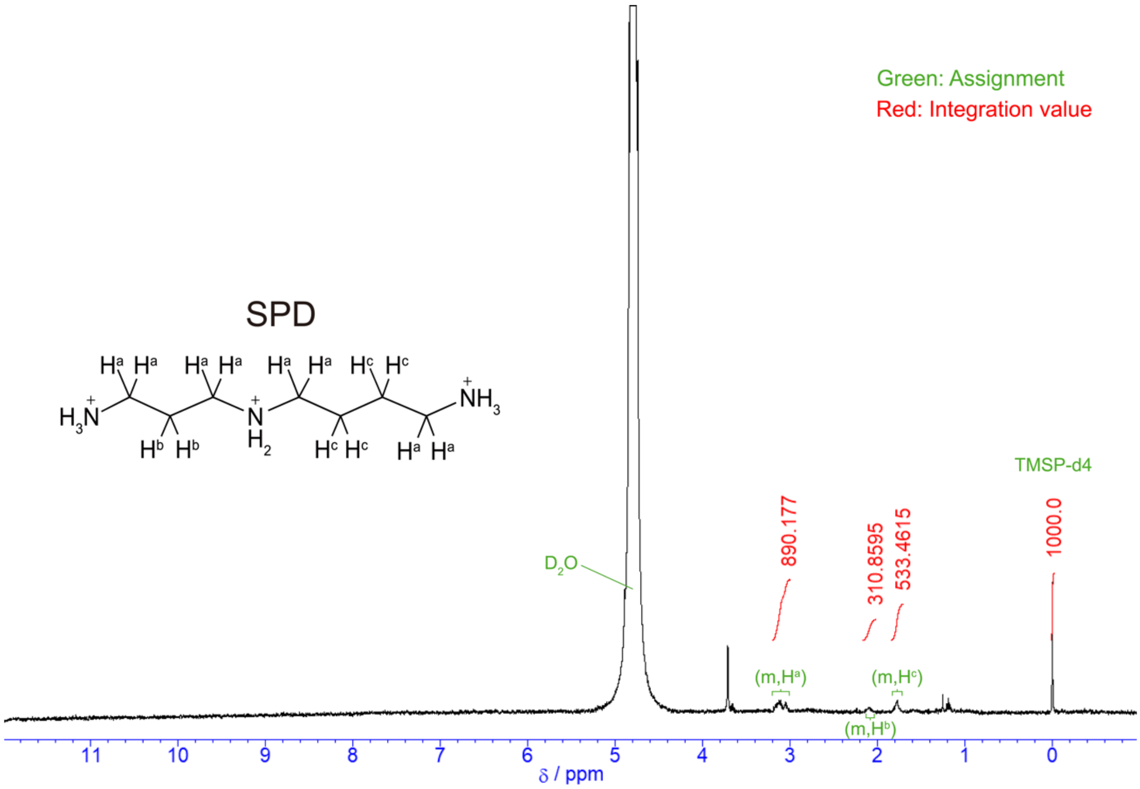

The chemical shift assignments of spermidine were performed manually by extracting spectral information from the HMDB [1].
(https://www.hmdb.ca/spectra/nmr_one_d/1671)

Spermidine: ^{1}H NMR (400 MHz, D_2O): δ 1.70-1.85 (m, 4H), 2.00-2.15 (m, 2H), 3.00-3.20 (m, 8H) ppm. CAS registry No: 124-20-9

(h) Enlarged spectra for each condition between 1.5 ppm and 3.5 ppm.

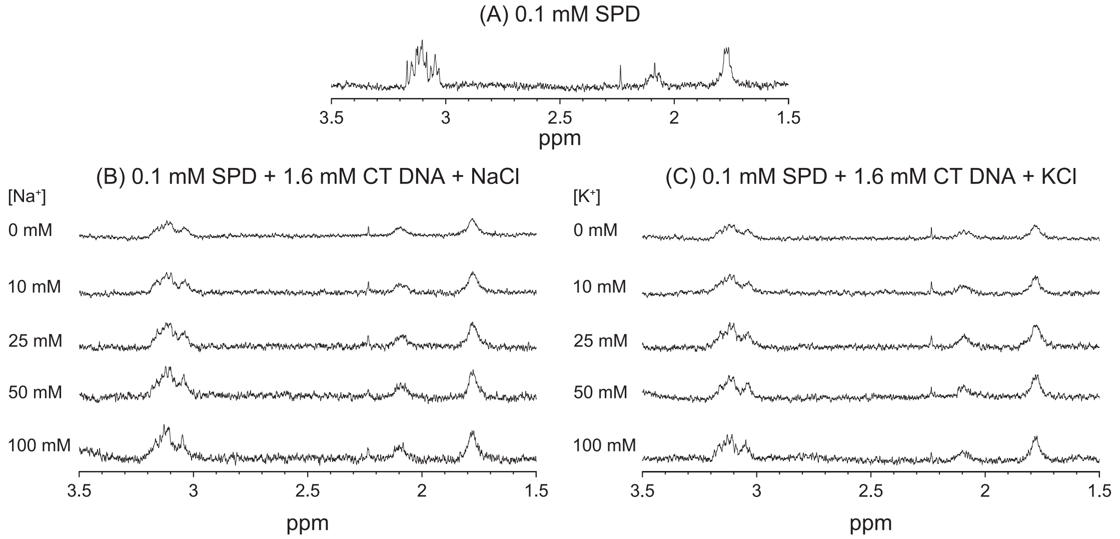

Reference

1. Wishart DS, Feunang YD, Marcu A, Guo AC, Liang K, Vázquez-Fresno R, et al., HMDB 4.0: The Human Metabolome Database for 2018. Nucleic Acids Res. 2018;46(D1):D608-D617.
